# Molecular and Biochemical Methods Useful for the Epigenetic Characterization of Chromatin-Associated Proteins in Bivalve Molluscs

**DOI:** 10.3389/fphys.2017.00490

**Published:** 2017-08-08

**Authors:** Ciro Rivera-Casas, Rodrigo Gonzalez-Romero, Rafael A. Garduño, Manjinder S. Cheema, Juan Ausio, Jose M. Eirin-Lopez

**Affiliations:** ^1^Environmental Epigenetics Group, Department of Biological Sciences, Florida International University North Miami, FL, United States; ^2^Department of Microbiology and Immunology, Dalhousie University Halifax, NS, Canada; ^3^Department of Biochemistry and Microbiology, University of Victoria Victoria, BC, Canada

**Keywords:** epigenetics, bivalves, chromatin, histones, SNBPs, methods, environment

## Abstract

Bivalve molluscs constitute a ubiquitous taxonomic group playing key functions in virtually all ecosystems, and encompassing critical commercial relevance. Along with a sessile and filter-feeding lifestyle in most cases, these characteristics make bivalves model sentinel organisms routinely used for environmental monitoring studies in aquatic habitats. The study of epigenetic mechanisms linking environmental exposure and specific physiological responses (i.e., environmental epigenetics) stands out as a very innovative monitoring strategy, given the role of epigenetic modifications in acclimatization and adaptation. Furthermore, the heritable nature of many of those modifications constitutes a very promising avenue to explore the applicability of epigenetic conditioning and selection in management and restoration strategies. Chromatin provides a framework for the study of environmental epigenetic responses. Unfortunately, chromatin and epigenetic information are very limited in most non-traditional model organisms and even completely lacking in most environmentally and ecologically relevant organisms. The present work aims to provide a comprehensive and reproducible experimental workflow for the study of bivalve chromatin. First, a series of guidelines for the molecular isolation of genes encoding chromatin-associated proteins is provided, including information on primers suitable for conventional PCR, Rapid Amplification of cDNA Ends (RACE), genome walking and quantitative PCR (qPCR) experiments. This section is followed by the description of methods specifically developed for the analysis of histone and SNBP proteins in different bivalve tissues, including protein extraction, purification, separation and immunodetection. Lastly, information about available antibodies, their specificity and performance is also provided. The tools and protocols described here complement current epigenetic analyses (usually limited to DNA methylation) by incorporating the study of structural elements modulating chromatin dynamics.

## Introduction

### Epigenetics and the structure of chromatin

In eukaryotes, DNA is packaged and compacted within the cell nucleus thanks to its association with chromosomal proteins, constituting the chromatin fiber. The fundamental subunit of the chromatin, the nucleosome, consists of approximately 145 bp of DNA wrapped around a protein core formed by small and highly basic proteins known as histones (H2A, H2B, H3, and H4 families) (Kornberg, 1974; van Holde, 1988). Higher-order chromatin structures are formed by the incorporation of linker histones (H1 family) which bind to adjacent nucleosomes and linker-DNA, facilitating the compaction of the chromatin fiber (Simpson, 1978). Histone proteins can be classified in two groups based on structural and functional considerations: canonical histones, which are incorporated to the DNA behind the replication fork; and histone variants, a group of functionally specialized proteins that are incorporated independently of the DNA synthesis. The dynamic exchange of histones in nucleosomes genome-wide may result in heritable (i.e., epigenetic) alterations in chromatin structure, regulating the accessibility of DNA for transcription, replication and repair factors involved in DNA metabolism (Wang et al., 2007b). Additionally, the chemical post-translational modifications (PTMs) of histone tails (e.g., phosphorylation, acetylation, methylation, etc.) can also modulate local chromatin environments, contributing to the epigenetic regulation of cell's responses to environmental changes (Wang et al., 2007a). The structural complexity and diversity of chromatin components is mirrored by its configuration in different cell types, especially in the case of the male germinal line. There, histones are almost completely replaced by smaller and even more basic proteins known as Sperm Nuclear Basic Proteins (SNBPs) (Ausio et al., 2007). These proteins can be divided into three types, evolutionary related to the linker histone H1, including: histone (H) type, protamine-like (PL) type, and protamine (P) type (Eirin-Lopez and Ausio, 2009). Overall, the study of germ chromatin structure and function is indispensable to understand how epigenetic marks are trans-generationally inherited.

### Histones and environmental epigenetic responses

Epigenetics constitutes the next frontier for understanding how mechanisms of temporal and spatial control of gene activity work during transient acclimatory responses and long-term adaptations (Holliday, 1990; Feil and Fraga, 2012; Palumbi et al., 2014). To this end, it is fundamental to investigate the relationships between specific epigenetic marks and subsequent modifications in gene expression patterns, as well as the environmental factors triggering those epigenetic marks (Cortessis et al., 2012). The study of the epigenetic mechanisms mediating exposure-response relationships constitutes the basis for environmental epigenetic analyses (Baccarelli and Bollati, 2009; Bollati and Baccarelli, 2010), providing information about how different environmental factors influence phenotypic variation (Cortessis et al., 2012; Suarez-Ulloa et al., 2015; Etchegaray and Mostoslavsky, 2016) and a very innovative and powerful tool to study adaptation (Etchegaray and Mostoslavsky, 2016; Rey et al., 2016). Chromatin provides a framework for the study of such environmental epigenetic responses (Allis et al., 2007), as demonstrated by the role of histone variants in the epigenetic regulation of different processes. For instance, histones H2A.X, H2A.Z, macroH2A, and H3.3 are involved in the maintenance of genomic integrity during exposure to genotoxic compounds (Rogakou et al., 1998; Xu C. et al., 2012; Xu Y. et al., 2012; Luijsterburg et al., 2016; Gonzalez-Romero et al., 2017). Similarly, H2A.Z is involved in responses to thermal fluctuations (Kumar and Wigge, 2010) and salicylic acid-dependent immunity (March-Diaz et al., 2008) in Arabidopsis. Also, macroH2A participates in the regulation of ribosomal genes in response to seasonal changes in the carp *Cyprinus carpo* (Araya et al., 2010). The role of histone variants during epigenetic responses is best exemplified by the differentiation of specialized histones in different species, possibly helping them to cope with specific life conditions (Van Doninck et al., 2009; Rutowicz et al., 2015). Such diversification further supports the contribution of histone variants to the adaptive evolution of living organisms (Talbert and Henikoff, 2014). Lastly, histone proteins can contribute to environmental responses by way of their antimicrobial role, as the release of histones or fragments of histones to the extracellular medium contribute to defend the cell against pathogens such as bacteria or viruses (Poirier et al., 2014; Bachere et al., 2015).

### Bivalve molluscs as emerging model organisms in environmental epigenetics

Bivalve molluscs constitute a very important group of invertebrates present in a great variety of environments, encompassing both marine and freshwater species. In addition, their sessile and filter-feeding lifestyle makes them excellent sentinel organisms in environmental studies (Gosling, 2003; Suarez-Ulloa et al., 2015). That, combined with the availability of genome sequences for charismatic species such as the Pacific oyster *Crassostrea gigas* (Zhang et al., 2012), the Pearl oyster *Pinctada fucata* (Takeuchi et al., 2012), and the Mediterranean mussel *Mytilus galloprovincialis* (Murgarella et al., 2016), support these organisms as emerging model systems in environmental epigenetics. On one hand, DNA methylation analyses have been conducted in oysters, demonstrating the implication of this mechanism in the regulation of gene expression (Gavery and Roberts, 2010, 2013; Riviere et al., 2013; Olson and Roberts, 2014; Riviere, 2014; Wang et al., 2014; Li et al., 2015; Saint-Carlier and Riviere, 2015; Jiang et al., 2016; Tran et al., 2016) as well as in responses to environmental stressors (Gonzalez-Romero et al., 2017). On the other, our work has provided information about histone diversity and function in the somatic line within this group (Eirin-Lopez et al., 2002, 2004a; Gonzalez-Romero et al., 2008, 2009, 2012a,b), including the discovery of histone variants such as macroH2A (Rivera-Casas et al., 2016a) or H2A.Z.2 (Rivera-Casas et al., 2016b). In the case of the germinal line, the structural and compositional heterogeneity in the sperm chromatin of bivalves has been elucidated (Ausio, 1986), including the evolutionary mechanisms leading to the differentiation of SNBPs from somatic histone H1 (Ausio, 1999; Eirin-Lopez et al., 2002, 2004a,b, 2006a,b; Gonzalez-Romero et al., 2009).

Chromatin components are remarkably conserved among eukaryotic organisms. However, while the basic methodologies for their study can be applied to a great variety of species, certain considerations should be made when working with bivalve molluscs. Here, we present a series of protocols suitable for the genetic and biochemical characterization of chromatin-associated proteins of bivalve molluscs. The workflow described here includes guidelines for the isolation and characterization of histone and SNBP genes, as well as protocols for the extraction, purification and analyses of their protein products. Overall, this work aims to constitute a useful reference methodological tool for researchers interested in the study of chromatin in bivalve molluscs, fostering environmental epigenetic analyses in this group.

## Experimental methods

### Histone and SNBP gene isolation in bivalves

The increasing availability of “omic” data, especially in a great diversity of non-model organisms, is currently facilitating the development of genetic analyses in ecologically and environmentally relevant organisms. Bivalves are not an exception to this trend, with the complete genome of the Pacific oyster, *Crassostrea gigas* (Zhang et al., 2012) and draft or low-coverage genomes of other species such as the Pearl oyster, *Pinctada fucata* (Takeuchi et al., 2012) and the Mediterranean mussel, *Mytilus galloprovincialis* (Murgarella et al., 2016) already available, as well as genome projects from other species such as the Eastern oyster, *Crassostrea virginica* (Gomez-Chiarri et al., 2015). In addition, multiple transcriptomes from other bivalve species are also available facilitating gene and protein discovery as well as structure and functional studies (e.g., as of March 2017, 225 public SRA BioProjects encompassing 15 different orders and 112 bivalve species). However, many of those resources are not yet available in most non-model bivalves, still requiring traditional methods to obtain genomic sequences. The present section provides information on gene sequence isolation and amplification for the most widely studied histone variants (H2A.X, H2A.Z, macroH2A, and H3.3) and SNBPs, facilitating the characterization of new sequences in bivalve species for which genome information is lacking.

#### Histone variants

The design of PCR and qPCR primers for histone variants is quite often limited by the high level of similarity among histones within a given family (e.g., H2A). Consequently, it is recommended to incorporate at least part of divergent untranslated regions (UTR, usually specific from different variants) into primer design, minimizing unspecific amplifications. On the contrary, the use of primers annealing in coding regions is a good strategy for amplification of histone sequences in unexplored species, either through RACE or genome walking experiments, given the higher conservation of these regions. Accordingly, the design of such primers requires a more careful sequence analysis in order to avoid targeting regions where variants and canonical histones are similar, as the latter are far more abundant and tend to amplify easily in PCR experiments. For primer design we recommend using the Primer-BLAST tool (Ye et al., 2012), which includes the software Primer3 (Untergasser et al., 2012) and BLAST searches in selected databases to avoid regions that can cause non-specific amplifications. Table 1 shows primers designed using this software to amplify histone variants in bivalves annealing in UTR regions, following the indications discussed above (note that some of these primers can be efficiently used in multiple species). It is important to note that species belonging to the genus *Mytilus* possess at least two different H2A.Z isoforms with specialized roles [H2A.Z.1, which is structurally and functionally equivalent to H2A.Z from other bivalve species, and H2A.Z.2, which is has been specifically identified in *Mytilus* (Rivera-Casas et al., 2016b)] and that some species (i.e., *C. gigas*) have evolved additional H2A.X genes (unpublished work), which will have to be considered when performing PCR experiments. In addition, primers for canonical histones as well as primers annealing in coding regions suitable for RACE or genome walking experiments are indicated in Supplementary Tables 1, 2, respectively.

**Table 1:** PCR primers amplifying histone variant genes in bivalves.

**Gene**	**Primer**	**Sequence (5′–3′)**	**Annealing temp**.	**Amplicon size (bp)**	**Species**	**Accession numbers**
H2A.X	H2A.X31Fw	TAAACATCTTCGTCGCAGTAAGATC	51°C	496	*Mytilus* spp.^*^, Chlamys varia^*^	MF152783
	H2A.X31Rv	TGCAGACTGACAAATACACT				
H2A.Z	H2A.Z12Fw	GAAGAAATTATGGCTGGCGGTA	50°C	596	*Mytilus* spp.^*^, Chlamys varia^*^	MF152784
	H2A.Z12Rv	AATGAGTCCGAGATGAATGC				
H2A.Z.2	H2A.Z.2_Full_Fw	AGTGGACACACAAAAGCACAAC	62°C	468	*Mytilus* spp.^*^	KU350311
	H2A.Z.2_Full_Rv	TGAGATGTTTGTAAAAGCTGCC				
MacroH2A	mH2A_Full_Fw	ACATGTCAGCATTTGTAGGTCA	62°C	1175	*Mytilus* spp.^*^	KT894822
	mH2A_Full_Rv	TCTCCTTGAATGACCTTGTCCA				
H3.3	H3.3Fw2	TGAAGAAGAATAAGTCGTGAACCG	59°C	523	*Chlamys varia*	MF152785
	H3.3Rv2	TGACTTGCATGATCTGTAGAAATTG				

* *Primers were originally designed to amplify the indicated histone variants in M. galloprovincialis and successfully employed in other Mytilus species and C. varia using less stringent PCR conditions. Accession numbers for H2A.X, H2A.Z, and H3.3 correspond to C. varia sequences, submitted to GenBank as part of this work*.

As a general rule, these primers have been successfully employed in PCR experiments at a final concentration of 0.2 μM. For each pair of primers, the annealing temperatures are indicated in the tables, as well as the amplicon length that will serve to calculate the extension time employed in the amplification of each histone gene. For the complete setup of the PCR reaction and thermal profile, we recommend following the Taq DNA polymerase manufacturer's guidelines.

#### Sperm nuclear basic proteins

SNBPs include a diverse group of proteins, including bivalve mollusc Protamine-Like (PL) proteins. However, all of them share a common evolutionary origin that can be traced back to histone H1 (Eirin-Lopez et al., 2006b). Nonetheless, SNBPs are much more divergent than histones (the PL content varies extensively even among related species). The present work provides information on accession numbers for two PL-I sequence isoforms (AY626224 and AY626225) from the surf clam *Spisula solidissima*, as well as PL-II/PL-IV (DQ305038) and PL-III (DQ305039) sequences from the mussel *M. californianus*. In the case of *Mytilus*, the PL-II/PL-IV precursor is post-translationally cleaved resulting in two different protein byproducts, PL-II^*^ and PL-IV. In addition, primers employed by previous reports in genome walking experiments for the characterization of these SNBPs (Lewis et al., 2004; Eirin-Lopez et al., 2006b) are indicated in Supplementary Table 2.

#### Quantitative PCR (qPCR) amplifications

**Figure 1:**
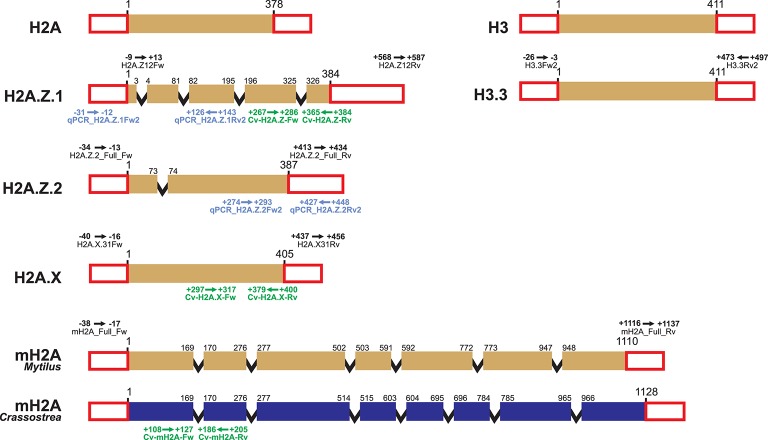
Schematic representation of bivalve histone gene structure and primer annealing positions. Histone genes H2A, H2A.Z.1, H2A.Z.2, H2A.X, macroH2A, H3, and H3.3 are represented using the mussel *Mytilus* as model species. Variations in length and intron/exon composition can be present in other bivalve species, such as the case of macroH2A in the oyster *Crassostrea*. Colored boxes represent coding exons, red uncolored boxes represent exons from UTR regions, and black lines represent introns (length not proportional to actual size). Details of PCR primers annealing in UTR regions (including name and annealing positions, see Table 1) are indicated above the corresponding histones. Details corresponding to qPCR primers (see Table 2) are indicated below each gene for the mussel *Mytilus californianus* (blue) and the Eastern oyster *Crassostrea virginica* (green).

Primer design for qPCR experiments is further constrained by the requirement of specific conditions, including small amplicon sizes (<200 bp), melting temperatures of approximately 60°C, 50–60% GC content, and low self-complementarity. However, since primers target gene regions displaying divergence among histones from the same family, it is not always possible to fulfill all requisites mentioned. Still, it is possible to design efficient qPCR primers for bivalve histone variants such as those shown in Table 2. Additionally, a good strategy to avoid amplification of genomic DNA in qPCR experiments is, when possible, to design primers annealing at intron/exon boundaries (Figure 1). Note that, although such positions are generally conserved across different species, some variation may be present in more divergent histone variants (i.e., macroH2A, Figure 1). Overall, the primers shown in Tables 1, 2, Supplementary Tables 1, 2, along with information displayed in Figure 1, provide a head start for the characterization of histone variants and SNBPs in bivalve species.

**Table 2:** Primers successfully employed in qPCR analyses of histone variant gene expression in bivalves.

**Gene**	**Primer**	**Sequence (5′–3′)**	**Annealing temp**.	**Amplicon size (pb)**	**Species**
H2A.X	Cv-H2A.X-Fw	AGTTACCATTGCCCAAGGAGG	60°C	104	*Crassostrea virginica*
	Cv-H2A.X-Rv	AAAATTCCTGGGACTGTGACGA			
H2A.Z	Cv-H2A.Z-Fw	CGCCATCAGAGGAGACGAAG	60°C	118	*Crassostrea virginica*
	Cv-H2A.Z-Rv	AGCTGTTTTCTGTGTGCCCT			
H2A.Z.1	qPCR_H2A.Z.1Fw2	TCGGTTGACCCAGTAATCCT	60°C	177	*Mytilus californianus*
	qPCR_H2A.Z.1Rv2	GCTCCTACTCGTCCATGACTT			
H2A.Z.2	qPCR_H2A.Z.2Fw2	AGAGGAGACGAGGAGTTGGA	60°C	175	*Mytilus californianus*
	qPCR_H2A.Z.2Rv2	TGAGCACTGTCAATGAGATGTT			
MacroH2A	Cv-mH2A-Fw	TCATTTCCGTATCGGAGCGG	60°C	98	*Crassostrea virginica*
	Cv-mH2A-Rv	CTCTTGCAGCATTTCCAGCC			

### Extraction of chromatin-associated proteins from bivalve molluscs

The extraction of histones and SNBPs is facilitated by their basic charge and high degree of conservation across eukaryotes. Consequently, standard isolation protocols using diluted acids can be easily implemented in a wide range of species. Nonetheless, extraction procedures still require modifications to account for specific particularities of different organisms, notably in the case of nuclei isolation, which can vary even among different tissues from the same species. The present work describes standardized protocols to analyze histone and SNBP content in different bivalve tissues (muscle, gill, hepatopancreas or digestive gland, hemolymph, male gonad, sperm, and female gonad), using the mussel *Mytilus* as a reference. These protocols have also been successfully applied to other bivalves, including clams (e.g., *Venerupis decussatus*; Gonzalez-Romero et al., 2008), oysters (e.g., *Crassostrea virginica*; Gonzalez-Romero et al., 2017) and scallops (e.g., *Chlamys varia*), supporting their efficiency across multiple taxonomic groups. Based on previous experience, gill, male gonad and sperm tissues produce good amounts of histone proteins following a standard acidic extraction protocol (Shechter et al., 2007). In the case of the remaining tissues (muscle, digestive gland, hemolymph, and female gonad), extractions can be optimized by incorporating the modifications detailed below (Gonzalez-Romero et al., 2012b, 2017; Rivera-Casas et al., 2016a,b).

#### Tissue collection and dissection

A starting material of 0.5 g of tissue (fresh or frozen) is enough for most experimental purposes. The collection of solid tissues (i.e., gills, digestive gland, muscle, or gonads) does not require special considerations other than the correct identification of such tissues, which can differ substantially among bivalve species. For instance, in the present context, the difference between male gonad and sperm refers to the developmental stage of the gonad. In other words, the sperm constitutes a very mature gonad whose content is mostly sperm cells. Thermal shock treatment (Soria et al., 2010), serotonin injections (Braley, 1985; Fong et al., 1996) or 0.5 M KCl injections (Breese et al., 1963; Beaumont et al., 1988) are required in order to get pure extracts of both sperm and eggs. In the case of liquid connective tissue (i.e., hemolymph), extractions can be performed *in vivo* by opening a small aperture in the shell using a lime (or a grinder if you work with a high number of individuals), reaching to the posterior adductor muscle using a hypodermic syringe. It is recommended to add Alsever's solution (2.05% glucose, 0.42% sodium chloride, 0.8% sodium citrate, and 0.055% citric acid) to the extraction syringe (500 μL of Alsever per every 10 mL of hemolymph) in order to avoid the hemocytes aggregation. Hemolymph must be collected carefully (preventing contamination from gonadal content), immediately transferred into 15 mL tubes and placed on ice until all samples are collected. Samples should be subsequently centrifuged at 250 × g for 5 min at 4°C, obtaining a pellet of bivalve hemocytes that are suitable for the subsequent processes.

#### Nuclei isolation

As a general rule, tissue is initially homogenized with a dounce homogenizer in 5 volumes of Buffer A (0.15 M NaCl, 10 mM Tris-HCl [pH 7.5], 0.5% Triton X-100) mixed with protease inhibitors 1/100 (v/v) (e.g., complete protease inhibitor cocktail [Roche Applied Science]). The homogenate is then placed on ice for 10 min (breaking cellular membranes), and centrifuged at 4,000 × g for 10 min at 4°C. In order to maximize yield and quality, this protocol should be modified depending on the tissue source as follows: In the case of digestive gland and muscle tissue, it is recommended to repeat homogenization steps at least once, as the insoluble materials contained in fat cells, plus the shape and composition of muscle cells, hamper cell membrane break and cell disaggregation, respectively. In the case of hemocytes, these steps should be repeated up to three times to obtain higher protein yields, due to the small size of these cells and their tendency to form aggregates obstructing disruption of cell membranes. Lastly, in the case of female gonad, a double amount of protease inhibitors should be used while skipping the incubation on ice for 10 min, in order to avoid the degradation of histone proteins as a result of the high content in proteolytic enzymes of this tissue.

Upon centrifugation, the pellet containing the nuclei is subjected to a second round of homogenization in 5 volumes of buffer without detergent, Buffer B (0.1 M KCl, 50 mM Tris-HCl [pH 7.5], 1 mM MgCl_2_), adding a mix of protease inhibitors 1/100 (v/v). The homogenate is then incubated on ice for 10 min and centrifuged at 4,000 × g for 10 min at 4°C. Once more, specific modifications are required for different tissue types. For digestive gland and muscle, prior to the homogenization in Buffer B, it is recommended to remove insoluble materials (present in high amounts in both tissues) by filtering nuclear extracts through a sterilized cheesecloth soaked in Buffer B. In the case of female gonad, doubling the amount of protease inhibitors and skip the incubation on ice for 10 min is recommended, in order to avoid the degradation of histone proteins by proteases. Overall, the nuclear fraction obtained at this point can be used in downstream experiments such as Micrococcal Nuclease (MNase) chromatin fractionation (Rivera-Casas et al., 2016a).

#### Histone and SNBP extraction and precipitation

The pelleted nuclear fraction constitutes the starting material for the extraction of bivalve chromosomal proteins. Accordingly, the supernatant resulting from treatment with Buffer B is discarded, the pellet resuspended in 2.5 volumes of 0.6 N HCl using a dounce homogenizer or by pipetting (both histones and SNBPs are very soluble in diluted acids such as 0.6 N HCl, due to their high basic amino acid content), and subsequently centrifuged at 8,200 × g for 10 min at 4°C. The resulting supernatant will be then transferred to fresh tube with 6 volumes of acetone, mixing the solutions by inverting the tubes 6-8 times, and incubating the samples at −20°C for at least 1 h. The solution will turn cloudy after a few minutes due to the precipitation of histones and SNBPs in acetone. In the case of tissues producing low protein yields (i.e., digestive gland, muscle or hemolymph), it is recommended that acetone incubation is extended overnight. Upon protein precipitation, samples are pelleted by centrifuging at 10,000 × g for 10 min at 4°C, discarding the supernatant and carefully washing the pellet with acetone in order to remove rests of acid. Samples are then subject to an additional centrifugation, carefully discarding the supernatant and air-drying the histone pellet for 20–30 min. Alternatively, a vacuum concentrator can be employed for 3–5 min (especially in the case of larger pellets). Lastly, proteins are dissolved in a variable volume of ultrapure water (depending on pellet size, 100 μL is appropriate in most cases), being ready to be used immediately or stored at −80°C. The integrity and concentration of histones and SNBPs can be assessed through protein separation in sodium dodecyl sulfate polyacrylamide gel electrophoresis (SDS-PAGE) and/or by using western blot experiments (see sections below).

**Figure 2:**
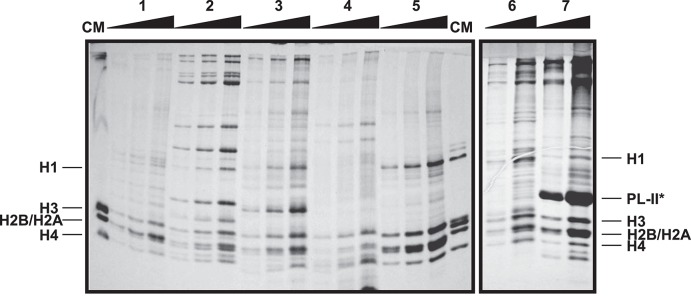
Electrophoretic analysis of histone and SNBP acid extractions from different tissues of the mussel *Mytilus californianus*. SDS-PAGE of histone extracts from hepatopancreas (1), muscle (2), male gonad (3), hemolymph (4), and gill (5), and histone and SNBP extracts from female gonad (6) and sperm (7). CM, chicken erythrocyte histones used as molecular marker. Band corresponding to PL-II^*^ in sperm tissue was identified based on our previous work (Gonzalez-Romero et al., 2012b). For each tissue, pellet resulting from the protein acid extraction (from 1 g of starting material) was resuspended in 100 μL of ultrapure-water, diluted in the same amount of SDS Sample Buffer and loaded 1, 2 and 4 μL in the SDS gel. Gradients in the upper part of the gel indicate the increase in the relative concentration of the proteins loaded in each lane.

The protein yield resulting from extractions will vary depending on the source tissue (Figure 2). For instance, protein extractions from gills provide the best results among somatic tissues in terms of purity and amount of protein. Digestive gland and hemolymph also provide relatively pure extracts when using the specific protocol modifications discussed previously. On the contrary, protein extracts from muscle tissues usually contain unidentified proteins in higher amount than histone proteins, reducing purity. In the case of germinal tissues, sperm extractions provide the highest amount of histones, due to the small size of the sperm cells. It is important to note that bivalve sperm chromatin, in addition to histones, is mostly composed by protamine-like proteins (increasing the compaction of DNA), constituting the most abundant proteins in SDS-PAGE gels (see band corresponding to mussel PL-II^*^ in Figure 2). Nonetheless, it is recommended to run acid-urea (AU) gels in order to discriminate the different PL types (see next section for details). In the case of male gonads is easy to obtain a good histone extract with no special considerations. However, depending on the developmental stage, the presence of PL proteins from sperm cells can be difficult to avoid. Lastly, it is important to avoid degradation of histone proteins when working with female gonad tissue. For that purpose, the implementation of the protocol outlined above yields a very good amount of histones without noticeable degradation.

### Histone and SNBP fractionation in bivalves

**Figure 3:**
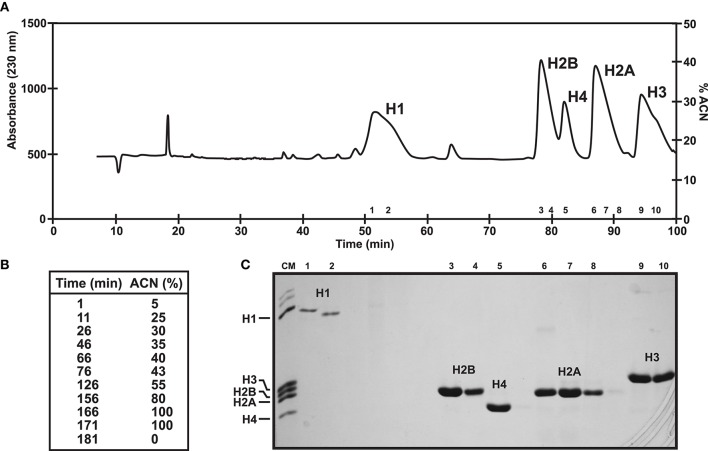
Histone fractionation in bivalves. **(A)** RP-HPLC chromatogram of 1 mg of histones from male gonad of the variegated scallop *Chlamys varia* using a *Vydac C18* column (300 Å pore diameter). Peaks indicate the elution of the five histone families. **(B)** Acetonitrile gradient employed in this experiment. **(C)** SDS-PAGE separation of selected fractions corresponding to each peak. CM, chicken erythrocyte histones used as molecular marker.

**Figure 4:**
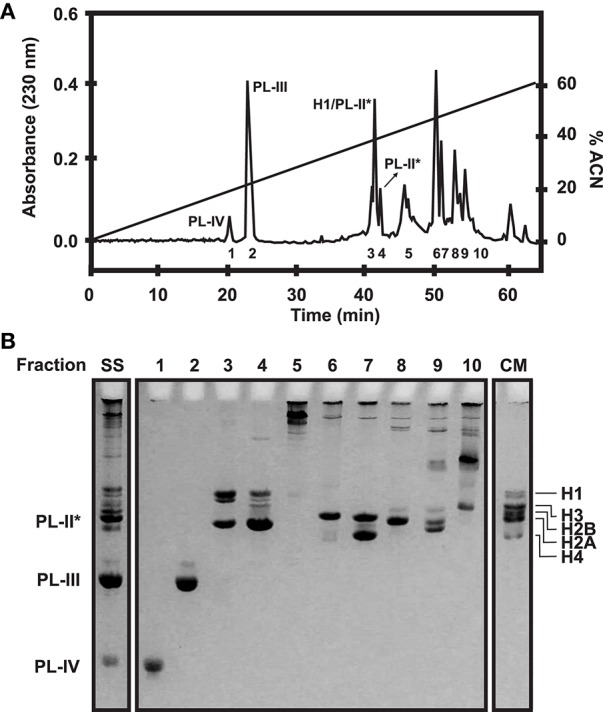
SNBP fractionation in bivalves. **(A)** RP-HPLC chromatogram of 1 mg of histones and SNBPs from *Mytilus* male gonad HCl extract using a *Vydac C18* column (300 Å pore diameter). Peaks corresponding to the different SNBP components are indicated in the figure. The acetonitrile gradient employed in this experiment is indicated by a straight line (0–60%). **(B)** AU-PAGE separation of selected fractions corresponding to each peak. SS, starting sample; CM, chicken erythrocyte histones used as molecular marker.

Chromosomal proteins can be easily purified using chromatographic approaches. More specifically, histones and SNBP extractions can be further purified using Reverse Phase High Performance Liquid Chromatography (RP-HPLC) (as described in Rocchini et al., 1995; Ausio and Moore, 1998; Shechter et al., 2007; Gonzalez-Romero et al., 2012b) and the resulting products can be employed in additional experiments such as nucleosome reconstitutions. The mobile phase (acetonitrile) gradient more often employed in separations, along with the corresponding chromatograms for histones and SNBPs are shown in Figures 3, 4, respectively. For detailed guidelines about RP-HPLC experiments for histone purification, see the work from Cheema and Ausio (2017).

**Figure 5:**
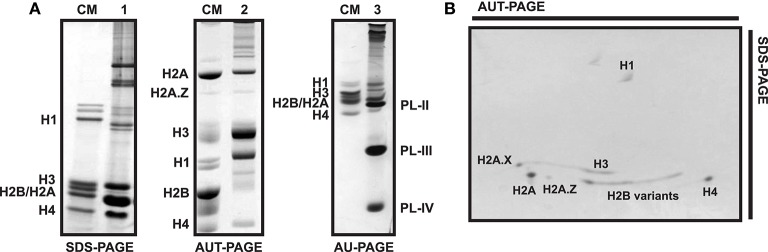
Electrophoretic profile of histone and SNBP proteins in different types of polyacrylamide gels. **(A)** SDS-PAGE (1) and AUT-PAGE (2) of male gonad from the variegated scallop *Chlamys varia*, AU-PAGE (3) of sperm from the mussel *Mytilus*. CM, chicken erythrocyte histones used as molecular marker. **(B)** Bi-dimensional PAGE from *C. varia* male gonad histone extract, being the first dimension an AUT gel and the second dimension an SDS gel. Bands corresponding to each histone or SNBP are indicated in the figures.

For analytical purposes, acid-extracted histones and SNBPs can be separated and analyzed using polyacrylamide gel electrophoresis under denaturing conditions such as SDS-PAGE (15% acrylamide, 0.4% bis-acrylamide), AU-PAGE (15% acrylamide, 0.1% bis-acrylamide, 5% acetic acid, 2.5 M urea), triton AU(AUT)-PAGE (10% acrylamide, 0.5% bis-acrylamide, 5% acetic acid, 5.25 M urea, 5 mM Triton X-100) or two-dimensional (2D)-PAGE (AU or AUT first dimension, SDS second dimension). Standard protocols for these gels can be used for the analysis of histones, although some modifications are recommended (see protocols in Box 1). The mobility patterns of histones and SNBPs in PAGE gels are indicated in Figure 5.

Box 1 **Protocols for polyacrylamide gel electrophoresis under denaturant conditions.**

**Table:** 

**SDS-PAGE (for 2 gels)**			**Sample buffer (2X)**	
**Reagent**	**Separating (15%)**	**Stacking (6%)**	**Reagent**	**Vol./W**
30%:0.8% acrylamide:bisacrylamide	5 mL	0.8 mL	0.5 M Tris pH 6.8	2.5 mL
1.5 M Tris pH 8.8	2.5 mL	x	10% SDS	4 mL
0.5 M Tris pH 6.8	x	1 mL	100% glycerol	2 mL
10% SDS	0.1 mL	0.04 mL	β-mercaptoethanol	1 mL
dH_2_O	2.32 mL	2.12 mL	Bromophenol Blue	0.1 g
10% APS	56.7 μL	40 μL	dH_2_O	0.5 mL
TEMED	4.5 μL	4 μL

– Set up the plates and prepare the gels as indicated in the tables. APS and TEMED should be added immediately before pouring the gels (the addition of these compounds initiates the polymerization).
– Add APS and TEMED to the separating gel and pour it into the plate letting a 2–3 cm gap in the upper part. Equilibrate it with water or isopropanol.
– Once the separating is solidified, remove the water or isopropanol, add APS and TEMED to the stacking gel and pour it introducing the comb immediately after.
– Dissolve the histone extract in the same volume of 2X SDS Sample Buffer as indicated in the table and boil the samples at 100°C for 2–3 min.
– Run the gel at 100 V for 1 h 30 min or until the dye band reaches the end of the gel.

**Running buffer (10X)**: 30.3 g of Tris Base, 144 g of glycine, 10 g of SDS, up to 1 L of dH_2_O.

**Table:** 

**Acetic-urea-triton gel**		**Acid-urea gel**	
**Reagent**	**Vol./W**	**Reagent**	**Vol./W**
Thiourea	7 mg	Thiourea	7 mg
20%:1% acrylamide:bisacrylamide	5 mL	30%:0.2% acrylamide:bisacrylamide	4 mL
Glacial acetic acid	480 μL	43% glacial acetic acid	1 mL
Urea (ultrapure)	3 g	10 M urea	2 mL
————————–Vortex————————-		dH_2_O	1 mL
45mM NH_4_OH	24 μL	30% H_2_O_2_	45 μL
25% Triton X-100	118 μL		
dH_2_O	1.33 mL		
30% H_2_O_2_	45 μL

– Set up the plates and prepare the gel as indicated in the tables. Pour the gel into the plate and insert the comb very quickly as these gels polymerize very quickly once you add 30% H_2_O_2_.
– Dissolve the histone/SNBP extract in the same volume of **2X Urea Sample Buffer** (4.8 g Urea, 1 mL glacial acetic acid, 20 mg Pyronin Dye Y, up to 10 mL dH_2_O).
– Run the gel at 100 V for 3 h 30 min to visualize histones in AU or AUT gels (or until the dye band reaches 2/3 of the gel) and for 2 h for the visualization of PLs in AU gels (for Mytilus spp.). **Running Buffer (1X)**: 5% acetic acid.
– ^*^Note that these gels run toward the negative pole, unlike SDS gels.

**Gel staining**

After the run, the staining procedure is the same for all the gels described above:

– Dissemble the gel and incubate it in **Staining Solution** (for 1 L: 100 mL glacial acetic acid, 250 mL isopropanol, 3 g Brilliant Blue, up to 1 L dH_2_O) for 1 h with constant shacking.
– Incubate in **Distaining Solution** (for 1 L: 100 mL glacial acetic acid, 250 mL isopropanol, up to 1 L dH_2_O) for 15 min with constant shacking and overnight with fresh Distaining solution.

Histones are separated based on their molecular weights in SDS gels. Consequently, while canonical histones H1, H3, and H4 can be easily isolated, it is more difficult to differentiate H2A and H2B due to their similar size (Figure 5A). Overall, SDS gels lack enough resolution to effectively discriminate among histones with similar molecular weights. That is especially evident in the case of histone variants differing only in a few residues from their canonical counterparts (e.g., H2A.Z.1, H2A.Z.2, H3.3) or histones bearing different PTMs (e.g., phosphorylation or acetylation), as they display almost identical mobility patterns. Other types of gels such as AU and AUT are therefore used to overcome these limitations, separating proteins based on their effective charge (unlike SDS, urea denatures proteins without affecting their charges). Furthermore, the addition of Triton X-100 to AUT gels allows the separation of histones also based on their hydrophobicity. More precisely, Triton increases the effective mass of proteins (except for the case of linker histones), consequently reducing their mobility (Waterborg, 2002). That is especially noticeable in the case of histones belonging to H2A and H3 families, which will now appear in the upper part of the gel (Figure 5A).

In addition to SDS and AUT gels, AU-PAGE constitute an important tool for the specific separation and analysis of SNBPs. These proteins often consist of a heterogeneous group of proteins whose composition varies even among related species (Ausio, 1999). For instance, the different PL components present in the sperm of mussels (PL-II^*^, PL-III, and PL-IV) can be perfectly separated using AU gels as depicted in Figure 5A. However, this pattern can vary in other bivalve species. Accordingly, the mature sperm chromatin in the surf clam *Spisula solidissima* is composed by a single PL type (PL-I), running in the upper part of AU gels due to its big size (>50 KDa). Similarly, additional separation of histone variants is possible using two-dimensional AUT-SDS gel electrophoresis. This approach provides higher resolution than any other gel type, allowing to differentiate H2A.X, H2A.Z, H3, and H2B variants (see Figure 5B). When coupled to other techniques such as western blot or mass spectrometry, two-dimensional gel electrophoresis constitute a powerful tool for the analysis of histones and their posttranslational modifications (Green and Do, 2009).

### Immunodetection of bivalve chromatin proteins

The specific detection of chromatin-associated proteins constitutes what is probably the most important challenge in chromatin and environmental epigenetic analyses in non-model organisms, motivated by the absence of species-specific antibodies. Both histones and SNBPs can be immunodetected after gel electrophoresis separation, using western blot experiments. Here, a SDS-PAGE western blot protocol to detect these proteins in bivalves is detailed. For western blot experiments from AU/AUT and two-dimensional gels, the reader should refer to the works by Shechter et al. (2007) and Green and Do (2009), respectively. Gel (SDS-PAGE) separation requires approximately 2 μg of protein extract (100 V for 1 h 30 min). Protein samples are subsequently transferred into a nitrocellulose membrane (100 V for 3 h at 4°C) in Transfer buffer (20 mM NaPO4 [pH 6.8], 14.25% ethanol, 0.1% SDS). In order to optimize the transfer, the membrane must be previously soaked in Transfer buffer for at least 20 min with constant shaking. Nitrocellulose membranes of 0.45 μm pore size are generally employed in histone immunodetection. However, if protein retention is suspected to be a problem, or the target histone is present at low levels, 0.2 μm pore size membranes should be used for better results, especially for smaller histones such as those belonging to the H2A (except for macroH2A), H2B and H4 families. In the case of larger histones such as H1 family and macroH2A variants, 0.45 μm membranes are recommended. Polyvinylidene difluoride (PVDF) membranes can also be used in these western experiments, following the specific preparation for those membranes.

Once transfer is completed, the gel is stained to verify protein migration into membrane, which is subsequently incubated in blocking buffer (PBS, 0.1% Tween, 3% powder milk) for 1 h at room temperature. That is ensued by the incubation of the membrane with a primary antibody at 4°C overnight, followed by three washing cycles in a PBS and 0.1% Tween solution at room temperature with constant shaking for 10 min. The membrane can be then incubated with a secondary antibody (recommended ECL Rabbit IgG, HRP-linked whole Ab, GE Healthcare) for 1 h at room temperature. This antibody is usually employed at 1:5,000 dilution in blocking buffer. The process is completed with three additional washing cycles as indicated above, proceeding to develop the blot. For that purpose, an enhanced chemiluminescent (ECL) system (such as ECL, Amersham biosciences) and visualization in X-ray films is recommended.

The evolutionary conservation of histone proteins enables, in some instances, the use of commercial antibodies (usually obtained from mammals including human, mice and rat) on invertebrates including bivalve molluscs. The present work provides information about the validity of commercial antibodies specific for different histone variants and PTMs in bivalves (see Table 3). All antibodies studied were raised in rabbits, although choices can be extended to every other available commercial antibody. Among H2A variants, H2A.X, and H2A.Z are relatively well conserved in metazoans, therefore, it is possible to use commercial antibodies for their immunodetection in bivalves. However, there are exceptions to this rule, best illustrated by the case of mussel H2A.Z. Two separate H2A.Z variants (H2A.Z.1 and H2A.Z.2), differing only in four residues, have been recently discovered in this organism, hindering their discrimination using commercial anti-H2A.Z antibodies (Rivera-Casas et al., 2016b). Commercial antibodies have also been successfully employed in immunodetection of bivalve H3 histones, including canonical histone H3 and H3S10 phosphorylation. It is important to note that, based on the antigenic peptides employed to obtain these anti-H3 antibodies (Table 3), they are expected to cross-react with histone variant H3.3 as well. In addition, since histone H3.3 is highly conserved across metazoans (e.g., bivalve H3.3 and human H3.3 proteins are identical), commercial antibodies should be suitable to detect this histone in most bivalve species, even though this awaits further analyses.

**Table 3:** Commercial and in-house developed antibodies detecting canonical histones and histone variants in bivalve molluscs.

	**Antibody**	**Commercial/Home-Made**	**Epitopes**	**Working dilution**	**Species**	**References**
**HISTONE**						
H2A.X	Anti-H2A.X	ABM (Y021260)	Q-A-SP-Q-E	1:3,000	*Mytilus*	Gonzalez-Romero et al., 2012b
	Anti-H2A.X	Home-Made	SQSQEF	1:3,000	*Mytilus*	Gonzalez-Romero et al., 2012b
	Anti-H2A.X	Abcam (ab47503)	Q-A-SP-Q-E	1:1,000	*Crassostrea virginica*	Gonzalez-Romero et al., 2017
	Anti-H2A.X pS139	Rockland (600-401-H36)	N/A (C-terminus)	1:1,000	*Crassostrea virginica*	Gonzalez-Romero et al., 2017
H2A.Z	Anti-H2A.Z	Abcam (ab4174)	N/A (C-terminus)	1:3,000	*Mytilus*	Gonzalez-Romero et al., 2012b
	Anti-H2A.Z	Thermo scientific (PA5-17336)	N/A (C-terminus)	1:1,000	*Crassostrea virginica*	Gonzalez-Romero et al., 2017
MacroH2A	Anti-MacroH2A	Home-Made (2 epitopes)	LSEKKLFLGQKM	1:1,000	*Mytilus*	Rivera-Casas et al., 2016a
			GGVLPHIHPELL			
H3	Anti-H3	Sigma (H0164)	IQLARRIRGERA	1:5,000	*Mytilus*	Unpublished
	Anti-H3	Rockland (100-401-E81)	N/A (C-terminus)	1:2,000	*Mytilus*	Unpublished
	Anti-H3 pS10	Millipore (09-797)	Amino acids surrounding PhosphoSer10	1:10,000	*Mytilus*	Unpublished
**SNBP**						
PL-II^*^	Anti-PL-II^*^	Home-Made	Whole protein	1:400 (Immuno-fluorescence)	*Mytilus*	Unpublished (see Figure 6)

The asterisk (^*^) in PL-II indicates that this protein is a cleavage product from the larger PL-II/PL-IV precursor (Carlos et al., 1993).

In the case of highly divergent histones such as macroH2A the availability of commercial antibodies suitable for bivalve molluscs is more improbable. This variant is much less conserved than H2A.X or H2A.Z, therefore, the amino acid variation can be significant even among macroH2As from closely related species. Thus, the only solution to this problem is developing a species-specific (or at least taxon-specific) antibody, as it was the case of the recently developed anti-macroH2A antibody suitable to detect this variant in a wide range of invertebrates including molluscs (Rivera-Casas et al., 2016a). However, since this antibody displays a small degree of cross-reactivity with histone H1 (a phenomenon also described in other anti-macroH2A antibodies from mammals, due to the structural similarities between both proteins; Pehrson et al., 1997), its application for genome-wide analyses such as chromatin immunoprecipitation (ChIP) is somewhat limited. Similarly, although commercial anti-H2A.X and anti-gammaH2A.X antibodies are suitable for bivalves, a bivalve-specific antibody developed in mussel is available for this variant, targeting the peptide SQSQEF characteristic from *Mytilus* H2A.X.

**Figure 6:**
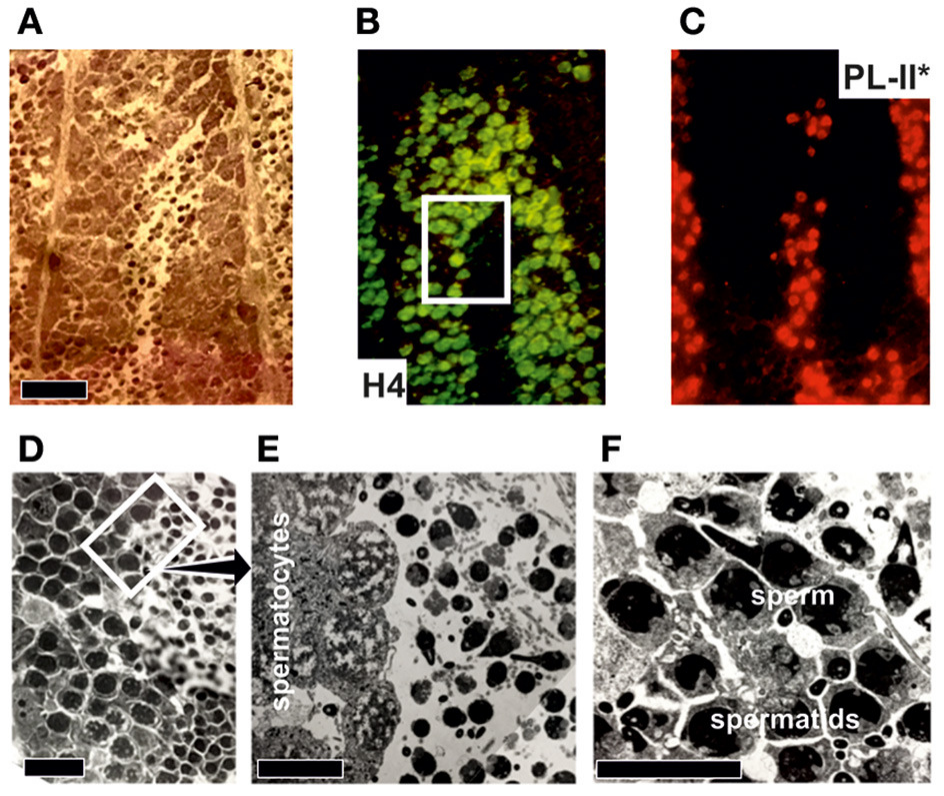
Immuno-histochemical analysis of PL-II^*^ distribution in male gonad from the mussel *Mytilus trossulus*. **(A)** Hematoxylin-eosin stained longitudinal section of the tip of a seminiferous tubule from a *M. trossulus* highly mature male gonad. The bar = 400 micrometers. **(B,C)** correspond to the same section shown in **(A)** but stained with histone H4 and PL-II^*^ primary antibodies respectively. The secondary antibodies were Oregon green for H4 and Rhodamine red for PL-II^*^. Notice the relatively low levels of histone H4 in spermatocytes compared to spermatids and sperm. **(D)** High magnification light microscope image of a section of the same tissue corresponding approximately to the square highlighted in **(B)**. The images were obtained as described in Casas et al. (1993). The bar = 200 micrometers. **(E,F)** represent electron microscopy images to show the three main different cell types present during the late spermiogenic stages of differentiation. The bars = 100 and 10 micrometers, respectively.

With the exception of an antibody for PL-II^*^ in the mussel *Mytilus* (Table 3), there have been no additional antibodies developed for SNBPs. Figure 6 provides an example for the immuno-histochemical use of this antibody. The protocol for the immunohistochemical procedure is detailed in Box 2. Overall, the high degree of conservation of most histone proteins enables the possibility of using commercial antibodies in bivalve tissues, overcoming one of the greatest limitations faced when working with non-model organisms.

Box 2 **Immunohistochemistry (IHC) protocol.**

**Fixing and embedding the tissue**

Dissect the gonads of mussel specimens and immerse them in primary fixative (4% freshly depolymerized paraformaldehyde in Millonig's Phosphate Buffer [11.04 g of NaH_2_PO_4_ in a total of 200 ml of deionized water, pH 7.4]). One group of gonads is kept uncut for light/fluorescence microscopy. Another group is cut into long 2-mm thick strips (it is important to create strips with different orientations). Fixation of both whole gonads and gonad strips is conducted for 1 h at room temperature.

Fixed specimens are then dehydrated in ethanol series (30% for 10 min—70% for 10 min—95% for 10 min—95% for 2 min − 100% for 5 min—100% for 5 min) using a volume of dehydrating solution of approximately 10-fold the volume of the tissue. Gonad strips are subsequently infiltrated with 50% LR-White (London Resin Co.) in 100% ethanol for 3 h, followed by pure LR-White resin overnight at room temperature. The whole gonads are treated with two steps of xylene for 1 h each at room temperature, followed by 6 steps of melted pure paraffin kept at 60°C for 20 min each. Gonads are then infiltrated in pure melted paraffin overnight.

Next day, LR-White embedded specimens are solidified in block molds at 60°C in a vacuum oven, as oxygen inhibits the polymerization of the LR-White resin. Paraffin-embedded specimens are then solidified in molds at room temperature. Paraffin blocks are kept at 4°C until sectioning.

**Sectioning and mounting the sections**

LR-White blocks are sectioned into 0.3–0.5 μm-thick sections in an ultramicrotome; while paraffin blocks are sectioned into ribbons of approximately 5 μm-thick sections in a histological microtome. Sections are subsequently mounted on glass slides, air dried and baked for 10 min at 50°C. Paraffin sections are then de-paraffinized in Copling jars using the following step series: xylene for 5 min (2 times)—100% ethanol for 5 min (2 times)—70% ethanol for 5 min (2 times)—Millonig's buffer for 5 min.

**Immunohistochemical staining**

Immunolabeling steps are performed at room temperature in Coplin jars, with the exception of antibody incubations, for which small volumes (50–150 μl, depending on the size of the sections) are used to cover the sections and the slides are kept in a humid chamber to prevent drying of the antibody solution during incubation. Briefly, sections are blocked in 1% bovine serum albumin −1% casein in Millonig's phosphate buffer for 1 h, washed once for 10 min in the same buffer, incubated with the primary antibody for 2 h, washed 3 times as before, incubated with the secondary antibody for 1 h, washed 3 times as before, blotted and mounted with No. 1 coverslips in gelvatol mounting medium (0.35 g Gelvatol, 3 ml Millonig's buffer, 1.5 ml glycerol) for observation. In this work, primary antibodies, house-made PL-II^*^ and H4 were diluted 1:400 and 1:200, respectively, in Millonig's phosphate buffer with 2% BSA; whereas commercial goat anti-rabbit IgG tagged with Rhodamine red (R6394) and Oregon green (O6381) from Molecular Probes (Eugene, OR) were diluted 1:200 in the same buffer.

**Histological staining**

After immunolabeling, sister sections are histologically stained to be used as reference for structural features of the immunolabeled sections. LR-White sections baked on glass slides (as above) are stained in a single-step procedure, covering the sections with freshly filtered Richardson's stain (prepared by mixing equal volumes of 1% azure II in deionized water, and 1% methylene blue in 1% borax), evaporating the stain to near dryness on a heating block at 50°C, and rinsing in deionized water to remove excess stain. De-paraffinized hydrated sections on glass slides (as above) are conventionally stained with Hematoxylin-Eosin. Histologically stained sections are permanently mounted with No. 1 coverslips in Permount™, before observation.

## Conclusions

The study of the mechanisms mediating physiological responses to environmental changes constitutes a key discipline to understand how climate change will affect organisms. Environmental epigenetics is at the center stage of such efforts, given the role of epigenetic modifications during the regulation of gene activity, and their implications for acclimatization and adaptation under ever-changing environments. Unfortunately, epigenetic information for most non-model and ecologically relevant organisms is very limited, with environmental epigenetic studies almost exclusively focused on DNA methylation (leaving other mechanisms largely unexplored). However, it is now clear that chromatin-associated proteins participate in organism-environment interactions in different capacities (e.g., regulation of gene expression, active role in defense against external pathogens, etc.). By providing a description of experimental methods for studying chromatin-associated proteins, the present work aims to provide a reference for researchers interested in studying how DNA is organized and regulated in molluscs, a ubiquitous taxonomic group playing critical functions in virtually all ecosystems. By doing so, this work fosters a more holistic approach to study the epigenetic mechanisms underlying environmental responses in bivalve molluscs, ultimately improving our understanding of their physiological responses to climate change, their application as environmental sentinel organisms as well as optimizing their management.

## Author contributions

The concept of the work was conceived by JE and developed in collaboration with CR, RG-R and JA. All authors were involved in the design and performance of the experiments as well as in the analyses of the data. CR and JE wrote the article, with contribution from RG-R and JA. All authors reviewed the manuscript draft.

### Conflict of interest statement

The authors declare that the research was conducted in the absence of any commercial or financial relationships that could be construed as a potential conflict of interest. The reviewer PI and handling Editor declared their shared affiliation, and the handling Editor states that the process nevertheless met the standards of a fair and objective review.

## Abbreviations

2D-PAGE: two-dimensional polyacrylamide gel electrophoresis
AU-PAGE: acid urea PAGE
AUT: acid urea triton PAGE
ChIP: chromatin immunoprecipitation
ECL: enhanced chemiluminescence
H: histone
MNase: micrococcal nuclease
P: protamine
PL: protamine-like
PTM: post-translational modification
PVDF: polyvinylidene difluoride
qPCR: quantitative PCR
RACE: rapid amplification of cDNA ends
RP-HPLC: reversed-phase HPLC
SNBP: sperm nuclear basic protein.
