# Supplementary material for: Molecular and Biochemical Methods Useful for the Epigenetic Characterization of Chromatin-Associated Proteins in Bivalve Molluscs
Source: Front Physiol. 2017 Aug 8;8:490. doi: 10.3389/fphys.2017.00490 (PMC5550673; doi:10.3389/fphys.2017.00490)
Supplement: Supplementary file 1 [file Table1.docx]

**SUPPLEMENTARY TABLES**

**Supplementary Table 1.** PCR primers amplifying canonical histone genes in bivalves.

| **Gene** | **Primer** | **Sequence (5' to 3')** | **Annealing temp.** | **Amplicon size (bp)** | **Species** | **Accession numbers** |
| --- | --- | --- | --- | --- | --- | --- |
| H1 | 5'-H1-full | TACCTGCGAAGACAATTCAG | 55.5 ºC | 1,100 | *Mytilus* spp.* | AJ416421- AJ416425 |
|  | 3'-H1-full | AGAAAGGGTAGGGCTCAG |  |  |  |  |
|  | H1 fwd | ACATATTCTGAAAGAAAAATTC | 53.5 ºC | 983 | *Solen marginatus* | FJ595834 |
|  | H1 rev | AGCAAGTACACATGGACTTT |  |  |  |  |
|  | 5′-H1 | GCGAAGACAATTCAGTCGGTT | 54 ºC | 1,064 | *Veneridae*** | EF670664;  EF670669;  EF670674 |
|  | 3′-H1 | GAAAGGGTAGGGCTCAGCT |  |  |  |  |
| H2A | 5′-H2A-full | ACTACCTGGAAGAAGCGAT | 52 ºC | 661 | *Mytilus* spp.* | AY267755- AY267759 |
|  | 3′-H2A-full | ACAGAGAAATGGAGGGAGT |  |  |  |  |
|  | H2A fwd | ACATTCAACCTAACTACCTG | 53.5 ºC | 728 | *Solen marginatus* | FJ595835 |
|  | H2A rev | TTCATTTTTTTCCCACCAACTATT |  |  |  |  |
|  | 5′-H2A | GGAAGAAGCGATGATTTGATTGG | 52 ºC | 643 | *Veneridae*** | EF670665;  EF670670;  EF670675 |
|  | 3′-H2A | GAGGGAGTGAGCTATGTTTGAG |  |  |  |  |
| H2B | 5′-H2B-full | GTCATTTTGGGGTGGGACACAG | 52 ºC | 663 | *Mytilus* spp.* | AY267740- AY267744 |
|  | 3′-H2B-full | CAAAACATCGCTTCTTCCAGGTAG |  |  |  |  |
|  | H2B fwd | TCCAGGTAGTTAGGTTGAATGTT | 53.5 ºC | 725 | *Solen marginatus* | FJ595836 |
|  | H2B rev | TAGGGGATGTATAGCACGAGAC |  |  |  |  |
|  | 5′-H2B | CATCGCTTCTTCCAGGTAG | 54 ºC | 657 | *Veneridae*** | EF670666;  EF670671;  EF670676 |
|  | 3′-H2B | TCATTTTGGGGTGGGACA |  |  |  |  |
| H3 | 5′-H3-full | TGTGTGCCAAATGTTAGCTTGG | 52 ºC | 878 | *Mytilus* spp.* | AY267745- AY267749 |
|  | 3′-H3-full | CAGTAACCTGACTGTCTTGGTCT |  |  |  |  |
|  | H3 fwd | GAACAATTGTTAGCTTCAA | 53.5 ºC | 811 | *Solen marginatus* | FJ595837 |
|  | H3 rev | TTTCTTCTTCTTTCAATACA |  |  |  |  |
|  | 5′-H3 | GGGGTGAACAATTGTTAGCTTC | 54 ºC | 858 | *Veneridae*** | EF670667;  EF670672;  EF670677 |
|  | 3′-H3 | TTCAGTAACCTGACTGTCTTGG |  |  |  |  |
| H4 | 5′-H4-full | ATTCCTACAGAGTTACCTCCCGGAT | 52 ºC | 601 | *Mytilus* spp.* | AY267750- AY267754 |
|  | 3′-H4-full | AAGTTGGACAAGTTGGACAGGAGA |  |  |  |  |
|  | H4 fwd | GAATTCCTACAGAGTTACC | 53.5 ºC | 612 | *Solen marginatus* | FJ595838 |
|  | H4 rev | TGTATCCACAGACTTGCTTGCC |  |  |  |  |
|  | 5′-H4 | CTACAGAGTTACCTCCCGGAT | 54 ºC | 591 | *Veneridae*** | EF670668;  EF670673;  EF670678 |
|  | 3′-H4 | ACAAGTTGGACAGGAGAAAGC |  |  |  |  |

**Mytilus* spp. includes the species *Mytilus galloprovincialis, M. trossulus, M. edulis, M. chilensis and M. californianus*.

**Veneridae includes the species *Ruditapes philippinarum*, *Venerupis decussatus* and *V. pullastra*.

**Supplementary Table 2.** PCR primers amplifying partial sequences of histone and SNBP genes and primers employed in RACE, genome walking and inverse PCR experiments in bivalves.

| **Gene** | **Primer** | **Sequence (5' to 3')** | **Amplicon size (bp)** | **Species** | **Accession numbers** |
| --- | --- | --- | --- | --- | --- |
| H2A | 5′-H2A-partial | AAGAGGTAAAAGTGGAAAGGCCCG | 366 | *Mytilus galloprovincialis* | - |
|  | 3′-H2A-partial | TAGCTTGATTTGCCGGTCTTCTTG |  |  |  |
| H2B | 5′-H2B-partial | CAAAGTCAACGGCACCCCGTG | 278 |  |  |
|  | 3′-H2B-partial | TTTGGCGAGTTCACCTGGCAG |  |  |  |
| H3 | 5′-H3-partial | TCGCAAATCTACAGGAGGGAAGGC | 340 |  |  |
|  | 3′-H3-partial | CATGATGGTAACCCTCTTGGCGTG |  |  |  |
| H4 | 5′-H4-partial | AAAGGAGGAAAGGGACTGGGA | 267 |  |  |
|  | 3′-H4-partial | CTGGCGTTTCAAGGCGTACAC |  |  |  |
| H2A.X | GSP1(Fw)-RACE | CGATCCTCCAGCAGCTTTCTTTGAC | **RACE** | *Mytilus galloprovincialis* | HQ242648 |
|  | NGSP1(Fw)-RACE | CACAGATCCCAATAGTCTATTAAG |  |  |  |
|  | GSP2(Rv)-RACE | TATCTTGCAGCAGAAGTGCTTG |  |  |  |
|  | NGSP2(Rv)-RACE | TCCAAGACATATACAACTAGCCATC |  |  |  |
| H2A.Z.1 | GSP1(Fw)-RACE | GTCTTCTGTGATCCCTTTTTGC | **RACE** | *Mytilus galloprovincialis* | HQ242649 |
|  | NGSP1(Fw)-RACE | ATGTGGAATGACACCACCACCA |  |  |  |
|  | GSP2 (Rv)-RACE | GGCGGTAAAGCGGGAA |  |  |  |
|  | NGSP2 (Rv)-RACE | AAGCTGTTTCTCGTTCCCAGA |  |  |  |
| H2A.Z.2 | H2A.Z.2 partial_Fw | CACCGCTGCCGTATACAGTGCCGCCATCTT | 232 | *Mytilus* spp | - |
|  | H2A.Z.2 partial_Rv | TCCTTGTGGACCTTTCTTCCCGATAA |  |  |  |
| Macro H2A | mH2A_Fw1 | TTGCAGCAACCCCAACAAAA | **RACE** | *Mytilus* spp | - |
|  | mH2A_Fw2 | AAAGCATCGGCTACTCCACC |  |  |  |
|  | mH2A_Rv1 | CCAGAACAAATGGCAGCACC |  |  |  |
|  | mH2A_Rv2 | GCTGATGTTGCTGCTACTGC |  |  |  |
| PL-I | SPISF3 | ATGATGAGCATGGTCGCTGCAGCCATTG | 306 | *Spisula solidissima* | - |
|  | SPISR2 | CATCGTCTTCTTTGTCTTCTTTGTGGTC |  |  |  |
|  | SPISGEN-F1 | GGCTCAGTAGGTTGGGTTCTTGTACC | **Genome walking** | *Spisula solidissima* | **Isoform a**  AY626224 |
|  | SPISGEN-F2 (nested) | GGGCAGCAAAGAGGTCCACAAAGAAGACCAC |  |  |  |
|  | SPISGEN-R1 | CATACTTGCGGATAGCTTGGGCTGAAGCACC |  |  | **Isoform b**  AY626225 |
|  | SPISGEN-R2 (nested) | CAATGGCTGCAGCGACCATGCTCATCAT |  |  |  |
| PL-III | MYTWKF1 | CAGCCTCCTCCCCCGGAAAGGCAGC | **Genome walking** | *Mytilus californianus* | DQ305039 |
|  | MYTWKF2 (nested) | CCAAAGAAAAGGAGGTCTGCTGGAAAG |  |  |  |
|  | MYTINV-F | GTCCTCATCACCAAAGAAAAGGAG | **Inverse PCR** | *Mytilus californianus* |  |
|  | MYTINV-R | CTTTCCCCTTCTTGGGGTCTTGGAAC |  |  |  |
